# FedADT: An Adaptive Method Based on Derivative Term for Federated Learning

**DOI:** 10.3390/s23136034

**Published:** 2023-06-29

**Authors:** Huimin Gao, Qingtao Wu, Xuhui Zhao, Junlong Zhu, Mingchuan Zhang

**Affiliations:** 1 School of Information Engineering, Henan University of Science and Technology, Luoyang 471023, China; gaomnli@haust.edu.cn (H.G.); zxh@haust.edu.cn (X.Z.); jlzhu@haust.edu.cn (J.Z.); zhang_mch@haust.edu.cn (M.Z.); 2 Intelligent System Science and Technology Innovation Center, Longmen Laboratory, Luoyang 471023, China

**Keywords:** federated learning, distributed training, stochastic gradient, derivative

## Abstract

Federated learning is served as a novel distributed training framework that enables multiple clients of the internet of things to collaboratively train a global model while the data remains local. However, the implement of federated learning faces many problems in practice, such as the large number of training for convergence due to the size of model and the lack of adaptivity by the stochastic gradient-based update at the client side. Meanwhile, it is sensitive to noise during the optimization process that can affect the performance of the final model. For these reasons, we propose Federated Adaptive learning based on Derivative Term, called FedADT in this paper, which incorporates adaptive step size and difference of gradient in the update of local model. To further reduce the influence of noise on the derivative term that is estimated by difference of gradient, we use moving average decay on the derivative term. Moreover, we analyze the convergence performance of the proposed algorithm for non-convex objective function, i.e., the convergence rate of $1/\sqrt{nT}$ can be achieved by choosing appropriate hyper-parameters, where *n* is the number of clients and *T* is the number of iterations, respectively. Finally, various experiments for the image classification task are conducted by training widely used convolutional neural network on MNIST and Fashion MNIST datasets to verify the effectiveness of FedADT. In addition, the receiver operating characteristic curve is used to display the result of the proposed algorithm by predicting the categories of clothing on the Fashion MNIST dataset.

## 1. Introduction

Recently, vast amounts of data have been generated by decentralized network edge devices, such as mobile phones and smart devices in the internet of things. Collecting and transmitting this data for training not only gives rise to network congestion, but also cause privacy leakage. For this reason, Federated Learning (FL) frameworks are proposed in [1,2], where clients learn a shared global model based on their own private data under the coordination of a central server. As data holders, clients conduct multi-step model training locally on the basis of the currently received global model, and then, the central server aggregates these local models, obtaining a global model, and returns it to each client. This manner of alternating training and communication was implemented by McMahan et al. [2], resulting in the Federated Averaging (FedAvg) algorithm, which is one of the most popular methods in federated learning. In FedAvg, each client trains a model by leveraging the Stochastic Gradient Descent (SGD) method. Due to its superiority, FL is broadly used in many application scenarios [3,4,5].

Despite the good empirical performance of FedAvg, also known as local SGD [6], there are still gaps between theory and practice in FedAvg. To better understand its convergence performance in theory, several studies [7,8,9] associated with it have emerged under data homogeneous setting in federated learning. For data or client heterogeneity, Ref. [10] introduced a regularization term to local object function settling the non-identically distributed challenge. Control variate and variance reduction methods [11] were proposed to correct the bias across clients, which leads to unstable and slow convergence. Most of these studies require extra communication cost or memory, which can be costly and unpractical in federated setting. In addition, momentum-based methods are introduced into either local model updates [12] or global model updates [13,14] or both [15,16] to improve the stability convergence. The convergence performance of SGD type methods are highly sensitive to the learning rate or step size that controls the speed of model learning; hence, another line of studies has sprung up aiming at modifying the learning rate, which scales the gradient for each dimension by incorporating prior information. These methods [14,17,18] that use adaptive learning rate also adopt the momentum term that accumulates the previous information. However, the accumulated past gradient information can cause an improper model update, which, sometimes, may be opposite to the descent direction. The training will be lagged and this lag effect can lead to an oscillation phenomenon in which the learning curve fluctuates at the optimal point, which is known as the overshoot problem in the domain of control.

In the optimization algorithms for machine learning, especially for deep learning, stochastic gradient can be regarded as an error where the goal of optimization is to make the error gradually settle to zero. This has a similar spirit as the Proportional Integral and Derivative (PID) controller in the fields of control theory and engineering. The main idea of the PID-based control method is incorporating the current, past, and the future information into the current correction to adjust the input of a dynamic system so it performs as desired. The use of a feedback mechanism makes the control process more responsive and robust [19,20]. The research [21,22,23] reveals that the error or deviation in the PID controller plays a similar role as the stochastic gradient used in SGD-based methods. In addition, SGD with momentum mainly utilizes current and historical gradients to optimize the model, which can be interpreted as the proportional and integral part. This inspires us to introduce the derivative information to the local update of federated learning, which denotes the future trend of the gradient change. According to the aforementioned analysis, we consider the descent direction and learning rate simultaneously in the update rule of model parameters for better training the federated learning model. To this end, this paper integrate PID controller into local SGD for federated learning. In a nutshell, the contributions are elaborated below:

- We incorporate the adaptive learning rate and derivative term in the update of the local model at the client side and propose a new federated optimization approach called FedADT.
- We rigorously prove that the proposed algorithm can achieve $O(1/\sqrt{nT})$ convergence rate for non-convex smooth objective functions, where *n* is the number of clients and *T* is the number of iterations.
- We conduct experiments for the image classification task on two datasets. The experiment results verify the effectiveness of the proposed algorithms.

The remainder is organized as follows. The related work is summarized in Section 2. In Section 3, the optimization problem is first introduced. Then, the proposed federated learning algorithm is described in detail in Section 4. In Section 5, we present related assumptions and the main results. The experiments are performed to validate the theoretical results in Section 6. Finally, we conclude the paper in Section 7.

## 2. Related Work

SGD is perhaps the most popular method, with good empirical performance in machine learning, which is also robust and scalable. Momentum is a heuristic, but a strong way to accelerate the convergence of SGD. Motivated by the heavy ball method [24] and Nesterov’s accelerate gradient method [25], a momentum term is usually added in the current update of descent directions by a weighed sum of previous information to improve the convergence of SGD [26]. Sutskever et al. [27] combined SGD with a careful use of the momentum method in the training of deep neural networks successfully. The article [28] developed the final iterate with standard step size schedules, and obtained the lower bounds for the sub-optimality of SGD. The generalization performance between SGD and a full gradient descent was developed by [29], and a novel separation result was presented in the stochastic convex optimization model. Additionally, adaptive optimization methods and variants [30,31,32] have gained fruitful achievements in deep learning because of their success in practice. Reddi et al. [32] proposed an Adaptive Mean Square Gradient method (AMSGrad) to amend the convergence issues of adaptive moment estimation method (Adam). Zaheer et al. [33] utilized the effect of a mini-batch size to improve the performance of Adam. The novel variant [34], which adapts step sizes according to the belief in current gradients (AdaBelief), has a better convergence, generalization, and training stability in both convex and non-convex cases by modifying Adam without additional parameters. Besides using a step size that adjusts to the scaling of gradients, a new class of adaptive methods [35,36,37] that is based on Polyak step size has emerged, utilizing both the current loss value and the stochastic gradient. Most of the studies only focus on the online convex optimization case or require projections operation on a bounded domain. Recently, the connection between PID control and stochastic optimization was described in [23], which shows that the PID-based method is an optimizer of encapsulating the gradient and momentum. An et al. [21] proposed a novel PID optimizer, which introduced derivative action to reduce the oscillation phenomenon, also known as overshoot in the control field. The above algorithms are implemented in a centralized setting.

Distributed optimization based on parallel SGD has been developed over the past decade, which often suffers from the bandwidth limits and large network delays. To alleviate communication bottlenecks, local SGD incorporating model averaging periodically results in the FedAvg algorithm [2], which significantly reduces the communication overhead. Along this line of research, there is much work that explores the theoretical convergence and improves the performance of FedAvg. Stich [6] firstly established the upper bound for FedAvg in a convex homogeneous setting when all clients participate at each round, and later it was improved by [8] in a convex heterogeneous setting. The work [38] established a lower bound for FedAvg in a heterogeneous case. Moreover, a unified framework [39] was presented to analyze local SGD methods in convex and strong convex settings. A hybrid local SGD method [40] was proposed to speed up the training of federated learning. These studies mentioned above use SGD as the local paradigm optimizer. One can also see other variants that incorporated momentum [9] and adaptive techniques [14,18]. FedAdam [14] utilizes Adam algorithm as a local optimizer in the federated learning framework to overcome the difficulty of parameters tuning for non-convex settings. Local AMSGrad [17] was designed to accelerate training and reduce communication overhead. Additionally, PID-based federated optimization methods have been developed recently. Ref. [41] designed a privacy budget allocation protocol by computing PID errors to balance privacy guarantee and the utility of the global model. The article [42] combined a federated learning framework and PID controller to develop the deployment of future intelligent transportation systems. Inspired by these works, in this paper, we use an adaptive learning rate and derivative term to the federated setting and analyze its convergence performance.

## 3. Problem Formulation

Notation. Throughout the paper, we use $x_{t}^{i}$ to denote the model parameter of *i*-th client at *t*-th iterations. Let ∥·∥ and ${∥\cdot ∥}_{\infty }$ be $l_{2}$ and $l_{\infty }$ vector norm, and ${\left(\cdot \right)}_{j}$ denotes the *j*-th coordinate of a vector. The vector square and vector division are element-wise, respectively.

**Figure 1:**
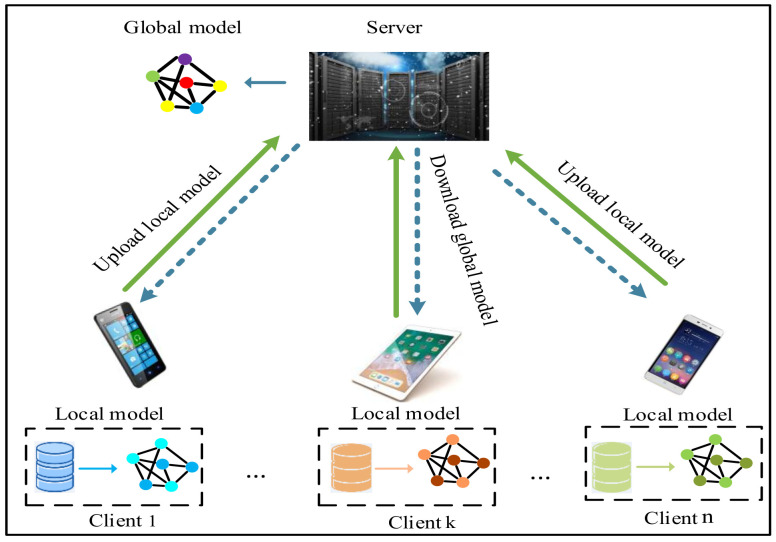
Federated learning system.

In this paper, we consider a general federated learning system, as shown in Figure 1. It contains *n* clients or devices and a central server; for example, a smart phone, industrial sensors. By collecting a large amount of production data, such as temperature, pressure, and current, federated learning can jointly model data from multiple plants without sharing trade secrets, thus improving productivity, quality, and safety. As illustrated in Figure 1, the training process of federated learning can be briefly summarized as follows: the central server firstly selected a subset of edge clients and a global model is downloaded by each client involved in the training at each round. Then, each client belonging to the subset begins multiple step local training based on its raw dataset and obtains a local model. Finally, the local models are uploaded and aggregated in the central server. The above process is repeated until the global mode converges or an expected predicted accuracy is attained.

In fact, the above model training can be modeled as an optimization problem. The main goal is to find a global model parameter, denoted by the vector $x\in R^{d}$, and the problem to be solved is formulated of the form:

(1) $$\begin{array}{ccc} \min_{x\in R^{d}}f\left(x\right) & = & \frac{1}{n}\sum_{i=1}^{n}f_{i}\left(x\right), \end{array}$$

where *d* is the dimension of model parameter, $f_{i}\left(x\right)=E_{\xi_{i}∼D_{i}}F\left(x;\xi_{i}\right)$ stands for the local expected loss function of *i*-th client, function $F(x;\xi_{i})$ denotes the loss for the model parameter x on one example $\xi_{i}$ stored in the *i*-th client, and $D_{i}$ represents the data distribution of *i*-th client, i∈{1,…,n}. For different clients, their data distribution may be different.

## 4. Algorithm Design

In this article, we are concerned with the collaborative learning of *n* clients under the coordination of a central server to solve problem (1) by local training and periodic model aggregation. In order to stabilize the process of local model training, we add a derivative term that denotes the trend of the gradient change and the adaptive learning rate to the update rule of the local model parameter. The pseudo-code of our proposed method, FedADT, is summarized in Algorithm 1. Specially, at the beginning of the (t+1)-th iteration, the central server random selects a subset of clients firstly. Each client *i* involved in current training computes the stochastic gradient $g_{t}^{i}$, which is an unbiased estimator of the full gradient $\nabla f_{i}\left(x_{t}^{i}\right)$, by using mini-batch random data from the dataset of the client *i*. Then, it computes an exponential weighted average momentum term $m_{t+1}^{i}$ as the descent direction of model update and second order moment $v_{t+1}^{i}$ to adaptively adjust the learning rate, respectively, which are defined as follows:

(2) $$\begin{array}{ccc} m_{t+1}^{i} & = & \beta_{1}m_{t}^{i}+\left(1-\beta_{1}\right)g_{t}^{i}, \end{array}$$

(3) $$\begin{array}{ccc} v_{t+1}^{i} & = & \beta_{2}v_{t}^{i}+\left(1-\beta_{2}\right)g_{t}^{i}⊙g_{t}^{i}, \end{array}$$

where $\beta_{1},\beta_{2}\in \left[0,1\right)$ are decay factors which control the exponential decay rates of weighted averages. In fact, $m_{t+1}^{i}$ can be expressed as $\left(1-\beta_{1}\right)\sum_{j=0}^{t}\beta_{1}^{t-j}g_{j}^{i}$ by recursion, where the initial value of momentum is set to 0. The decay factors $\beta_{1}$ are usually chosen so that the exponential weighted averages allocate small weights to previous gradients that are far from the current moment. A similar choice applies to the decay factor $\beta_{2}$, which is selected from the set {0.99, 0.9999} in the relevant papers [31,32]. Notation ⊙ indicates the element-wise square.

Then, a first difference term that suggests the future information is added to correct the lagged gradient. In fact, the differential of gradient is approximated by the first difference $g_{t}^{i}-g_{t-1}^{i}$, which reflects the instant variation of gradient. It is incorporated in the design of algorithm to exploit the future expectation of the model and avoid overshooting, which acts in a similar role as in the PID controller. Furthermore, in order to mitigate the noise in gradient calculation caused by randomly selecting mini-batch data, we use moving weighted average on the derivative part, resulting in:

(4) $$\begin{array}{ccc} d_{t+1}^{i} & = & \beta_{1}d_{t}^{i}+\left(1-\beta_{1}\right)\left(g_{t}^{i}-g_{t-1}^{i}\right). \end{array}$$

Finally, the local model parameter $x_{t+1}^{i}$ is updated, i.e.,

(5) $$\begin{array}{cc} \; & \left\{\begin{array}{c} \frac{1}{n}\sum_{i=1}^{n}\left(x_{t}^{i}-\frac{\eta }{\sqrt{{\hat{v}}_{t+1}}}⊙m_{t+1}^{i}+\nu d_{t+1}^{i}\right),t+1\;\mathrm{mod}\;E=0,\\ x_{t}^{i}-\frac{\eta }{\sqrt{{\hat{v}}_{t+1}}}⊙m_{t+1}^{i}+\nu d_{t+1}^{i},\;\mathrm{otherwise}, \end{array}\right. \end{array}$$

where η is learning rate, and ν is the step size of derivative term. The term ${\hat{v}}_{t+1}$ is the element-wise maximum of ${\hat{v}}_{t}$ and the average of $v_{t+1}^{i}$ across *n* clients, as shown in line 11 of Algorithm 1. Moreover, if t+1 is a multiple of *E*, the central server averages the model parameters $x_{t+1}^{i}$ and the second moment $v_{t+1}^{i}$, where *E* is a positive constant denoting the number of local updates.
**Algorithm 1** Federated Adaptive Learning Based on Derivative Term (FedADT)Input: Initial point $x_{0}$, $m_{0}^{i}=0$, ${\hat{v}}_{0}^{i}=\delta 1$, the number of iterations *T*1:fort=0,1,…,T−1do2:   forclienti=1,2,…,n in parallel do3:       Compute gradient: $g_{t}^{i}=\nabla f_{i}\left(x_{t}^{i},\xi_{t}^{i}\right)$4:       Update $m_{t+1}^{i}=\beta_{1}m_{t}^{i}+\left(1-\beta_{1}\right)g_{t}^{i}$5:       Update $v_{t+1}^{i}=\beta_{2}v_{t}^{i}+\left(1-\beta_{2}\right)g_{t}^{i}⊙g_{t}^{i}$6:       Update $d_{t+1}^{i}=\beta_{1}d_{t}^{i}+\left(1-\beta_{1}\right)\left(g_{t}^{i}-g_{t-1}^{i}\right)$7:       ift+1modE≠0then8:            ${\hat{v}}_{t+1}={\hat{v}}_{t}$9:            $x_{t+1}^{i}\leftarrow x_{t}^{i}-\frac{\eta }{\sqrt{{\hat{v}}_{t+1}}}⊙m_{t+1}^{i}+\nu d_{t+1}^{i}$10:       else11:            ${\hat{v}}_{t+1}=\max\left\{\frac{1}{n}\sum_{i=1}^{n}v_{t+1}^{i},{\hat{v}}_{t}\right\}$12:            $x_{t+1}^{i}\leftarrow \frac{1}{n}\sum_{i=1}^{n}\left(x_{t}^{i}-\frac{\eta }{\sqrt{{\hat{v}}_{t+1}}}⊙m_{t+1}^{i}+\nu d_{t+1}^{i}\right)$13:       endif14:   endfor15:endfor

## 5. Assumptions and Main Results

In this section, before providing the main results of Algorithm 1, we first state three assumptions as follows.

**Assumption** **1.** *The loss function $f_{i}\left(x\right)$ is differentiable and L-smooth, L>0 is a constant; that is, for $\forall x,y\in R^{d}$ and i∈{1,…,n}, we have:*

(6) $$∥\nabla f_{i}\left(x\right)-\nabla f_{i}\left(y\right)∥\leq L∥x-y∥.$$

Assumption 1 expounds our requirements for local objective functions, and is common in non-convex problems [14,17,43]. Next, there are two different assumptions about the stochastic gradients.

**Assumption** **2.** *The stochastic gradient $g_{t}^{i}$ has bounded $l_{\infty }$ norm, i.e., for any i∈{1,…,n} and t∈{1,…,T}:*

(7) $${∥g_{t}^{i}∥}_{\infty }\leq G_{\infty },$$

*where $G_{\infty }$ is a positive scalar.*

**Assumption** **3.** *The stochastic estimator $g_{t}^{i}\left(x,\xi \right)$ of full gradient $\nabla f_{i}\left(x\right)$ is unbiased, and its coordination-wise has σ-bounded variance, i.e., for any $x\in R^{d}$ and j∈{1,…,d}:*

(8) $$E\left[g_{t}^{i}\right]=\nabla f_{i}\left(x_{t}^{i}\right),$$

(9) $$E{\left|{\left(g_{t}^{i}\right)}_{j}-{\left(\nabla f_{i}\left(x_{t}^{i}\right)\right)}_{j}\right|}^{2}\leq \sigma^{2},$$

*where σ>0 is a constant.*

The two assumptions above are common in the analysis of adaptive-type methods [14,17], which bound the gradient estimate with noise and the variance of the stochastic gradient.

From the above assumptions, we obtain the main convergence theorem for the FedADT algorithm.

**Theorem 1.**  *Consider problem (1) under Assumptions 1–3, if learning rate η and step size ν satisfy $\eta =\min\{\frac{\sqrt{n}}{\sqrt{Td}},\frac{\sqrt{\delta }}{4L}\}$ and $\nu =\frac{1}{\sqrt{Td}}$, then for any $T\geq \frac{16nL^{2}}{d\delta }$, we have:*

(10) 1T∑t=0T−1E∇f(x¯t)v^t+11/42≤8dnTf(x¯0)−f∗+2dnTLσ2δ+8dTβ11−β1G∞2δ=+2dTβ12(1−β1)2G∞2δ+16nTL2G∞2δ3/2β12(1−β1)2+5E24=+16TL2G∞2δ5(1−β1)2β12E2+1,

*where ${\bar{x}}_{t}=\frac{1}{n}\sum_{i=1}^{n}x_{t}^{i}$, and $f^{*}≜f\left(x^{*}\right)$ is the optimal value at the optimal point $x^{*}$.*

We defer to the proof of Theorem 1 in Appendix A.

**Remark 1.**  *From Theorem 1, we can see that the convergence rate of FedADT mainly relies on the initial value of function and the variance of stochastic gradients and the number of local updates. The terms involving $\beta_{1}$ are introduced due to the use of momentum and derivatives. Moreover, the coefficient ${\left(1-\beta_{1}\right)}^{2}/\beta_{1}^{2}$, referred by derivative in the last term, is less than 1. In addition, the number of local update E affects both the communication efficiency and the convergence upper bound, which incurs the bias of decent directions by the local update. However, it is obvious that the terms containing E will not dominate on the right-hand side of (10) when $E\leq O(\frac{{\left(dT\right)}^{1/4}}{n^{3/4}})$. In fact, if T>nd, we can simplify the upper bound of (10) and achieve the convergence rate $O(\sqrt{d}/\sqrt{nT})$ for the proposed algorithm, as shown in Corollary 1.*

**Remark 2.**  *In addition, the worst case that the right-hand side of (10) can be large if δ is small will not happen. In fact, the term δ arises from the lower bound of ${\hat{v}}_{t}$, and together with the update rule, it will quickly become at least the same in the sense of order as second moment of the stochastic gradients. Additionally, the stochastic gradients can also be small, so their $l_{\infty }$ norm keeps in the order of δ.*

**Corollary 1.**  *Under Assumptions 1–3, let $E\leq O(\frac{{\left(dT\right)}^{1/4}}{n^{3/4}})$; for $T\geq \max\{\frac{16nL^{2}}{d\delta },nd\}$, we have*

$$\begin{array}{c} \frac{1}{T}\sum_{t=0}^{T-1}E{∥\nabla f({\bar{x}}_{t})∥}^{2}\leq O\left(\frac{\sqrt{d}}{\sqrt{nT}}\right). \end{array}$$

From Corollary 1, we can see that the convergence of the proposed algorithm is evaluated by $\frac{1}{T}\sum_{t=0}^{T-1}E{∥\nabla f({\bar{x}}_{t})∥}^{2}$, which is exactly the lower bound of the term on the left-hand side of (10) by utilizing the inequality ${∥{\hat{v}}_{t+1}∥}_{\infty }\leq G_{\infty }$.

## 6. Experiments

In this section, we study the performance of the proposed algorithm on two standard datasets for the image classifications task in a federated setting. The MNIST dataset [44] is a set of handwritten digits from 0 to 9 which belong to 10 different categories. It contains 60,000 training samples and 10,000 training samples. Each image is a 28×28 pixels grey, handwritten digital image with white text on a black background. Fashion MNIST [45] is a clothing image dataset which contains 10 classes of items such as T-shirt, dress, and bag. It has the same training and test samples as the MNIST dataset, which are summarized in detail in Table 1. We evaluate our algorithm FedADT on two datasets by training a Convolutional Neural Network (CNN) as in [2], which includes two convolution layers and two pooling layers followed by a fully connected layer with more than a million parameters in total.

In the experiments, we use 10 nodes and a central server to mimic the federated training setting. Each node trains a local model and uploads it to central server periodically. The central server generates a global model by aggregating local models. Here, the number of local updates is set to 5, and the local batch size is chosen from {5, 16, 64} to test for the best performance. We suppose each node takes part in the training at each communication round. We select the learning rate from {0.1,0.01,0.001,0.00001,0.00002} for the best performance, and set $\beta_{1}=0.9$, $\beta_{2}=0.99$. For baseline algorithms, we search the learning rate from the same range as above, i.e.,{0.1,0.01,0.001,0.00001,0.00002}. We set the local update number as 5 with a batch size of 16. For comparison purposes, we assume that each client has the same neural network model which is trained by different algorithms. In addition, we account for two ways of partitioning data over nodes as in [2], i.e., data homogeneity (IID setting), where the date is uniformly distributed to 10 nodes, and data heterogeneity (Non-IID setting), where the data is shuffled by digit label and then assigned to 10 nodes. Both of these divisions are balanced. All the experiments in the article are performed on a workstation with two Intel(R) Xeon(R) Silver 4114 CPUs and two NVIDIA GeForce GTX 1080 Ti GPUs, and the algorithms are implemented in PyTorch framework, which is a popular deep learning training library.

**Figure 2:**
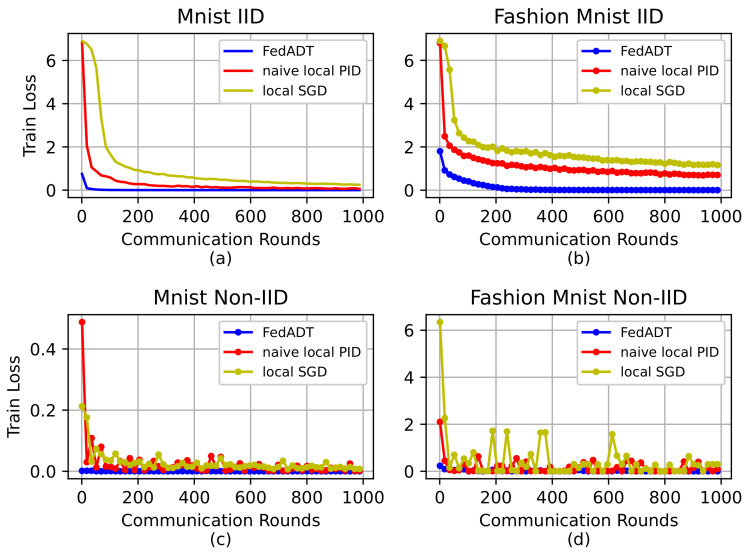
Training loss of FedADT and baseline algorithms under different data distributions. (**a**) MNIST with IID data. (**b**) Fashion MNIST with IID data. (**c**) MNIST with Non-IID data. (**d**) Fashion MNIST with Non-IID data.

We use two common metrics: training loss and test accuracy in federated learning and plot the learning curves as increasing communication rounds to verify the performance of different methods. We conduct 1000 rounds on two datasets to compare FedADT with naive local PID and local SGD methods, which use PID [21] and SGD as the local optimizers for each node. The results are illustrated in Figure 2, which exhibits the loss curves of three federated optimization algorithms over MNIST and Fashion MNIST datasets, respectively. We can see that the proposed FedADT method consistently achieves a faster convergence performance compared with the two baseline methods both under IID and Non-IID data setting of two datasets. However, for the bottom-row of Figure 2 with the data heterogeneity among different nodes, the learning curves of naive local PID and local SGD oscillate and are unstable, which slow down the convergence and the global models require more training rounds so as to obtain the desired results. A reasonable explanation is that FedADT uses the differential term in local decent direction, as well as the adaptive learning rate in the update process of the model.

**Figure 3:**
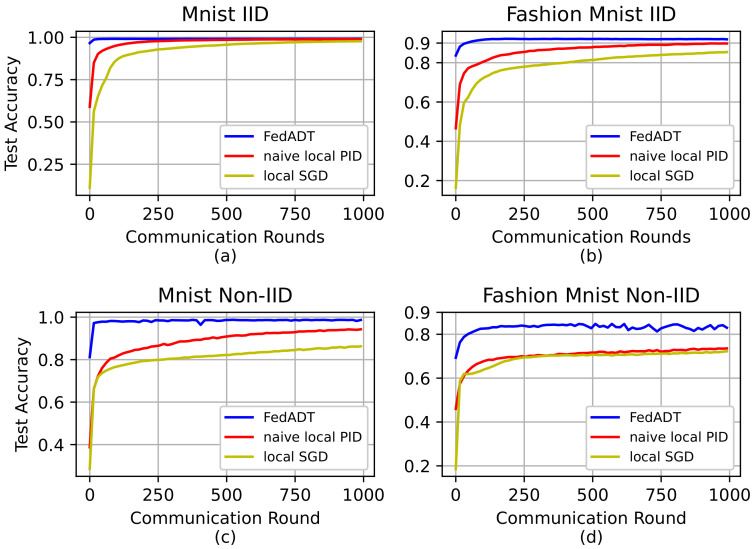
Test accuracy of FedADT and baseline algorithms under different data distributions. (**a**) MNIST with IID data. (**b**) Fashion MNIST with IID data. (**c**) MNIST with Non-IID data. (**d**) Fashion MNIST with Non-IID data.

Figure 3 shows the advantage of FedADT in terms of test accuracy under different data distributions over MNIST and Fashion MNIST datasets. As expected, all the algorithms can achieve almost similar accuracy in training the network under IID data setting on both datasets. Compared with the baseline methods, the proposed algorithm achieves the highest accuracy, 99.13% and 91.98%, first on the two datasets, respectively. For the data heterogeneity among different nodes, as shown in the bottom row of Figure 3, we have observed that the best accuracy of FedADT is 4.41% and 12.25% higher than that of naive local PID (94.37%) and local SGD (86.53%) on the MNIST dataset, respectively. On the Fashion MNIST dataset, the best accuracy of FedADT is 10.87% and 12.65% higher than that of naive local PID (74.02%) and local SGD (72.24%), respectively.

**Figure 4:**
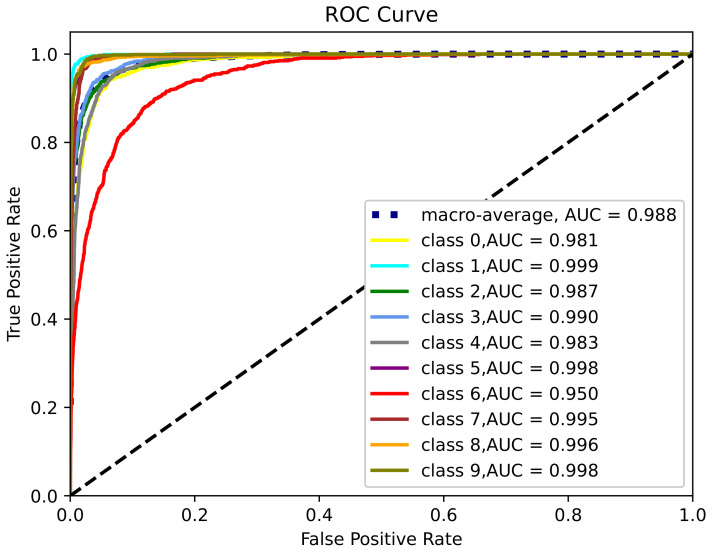
The ROC curve of classification with Non-IID data on Fashion MNIST dataset.

We use Receiver Operating Characteristic Curve (ROC) and Area Under Curve (AUC) as evaluation standards for the quality of the proposed algorithm. The experiment is performed by training the convolutional neural network on the Fashion MNIST dataset with Non-IID data, and the experimental result is shown in Figure 4. It demonstrates the characteristic curve and AUC area for 10 different clothing items. We can observe that class 1 is best identified and has the biggest AUC value of 0.999, and class 9 and 5 have similar results as class 1, whereas class 6 is not well recognized, and has the smallest AUC value, 0.950.

## 7. Conclusions

In this paper, we focus on the federated learning, where multiple nodes or clients are jointly modeled without exchanging their privacy data. During the local model training stage, the client side generally adopts a stochastic gradient-based algorithm, which is sensitive to step size and suffers from slow convergence performance. Inspired by the control theory, we first propose a federated adaptive learning method based on the derivative term which acts in a similar role as in the PID controller. We utilize the first difference of gradient to estimate the derivative term, which reflects the instant variation of gradient and stands for the future information. We provide a convergence guarantee for our proposed algorithm; in particular, when $E\leq O(\frac{{\left(dT\right)}^{1/4}}{n^{3/4}})$ and T>nd, the convergence rate of $O(1/\sqrt{nT})$ is achieved for non-convex objective functions. Finally, the experiments are performed under different data distribution cases on MNIST and Fashion MNIST datasets. Specially, for the Fashion MNIST dataset, the training loss curve of our proposed algorithm declines fastest compared to other baseline methods, and the highest accuracies of 91.98% and 84.89% are attained under IID and Non-IID cases, respectively. Similar results are obtained on the MNIST dataset, which empirically verify the effectiveness of the proposed algorithm. In addition, the ROC curve is used to display the satisfactory result by predicting the categories of clothing on the Fashion MNIST dataset. In future work, we will consider the effective-communication mechanism without sacrificing the convergence rate. At the same time, privacy protection should be considered in the process of communication between clients and the central server.

## Figures and Tables

**Table 1:** Summary of datasets.

Name	Training	Testing	Class
MNIST	60,000	10,000	10
Fashion MNIST	60,000	10,000	10

## Data Availability

Not applicable.

## Appendix A. Proof of Theorem

In this section, we analyze the convergence of the proposed algorithm following the method [9,17,18] which helps to handle the stochastic momentum term. Along this line, a virtual sequence is often useful for theoretical analysis.

### Appendix A.1. Main Proof of Theorem

Before providing the main proof of Theorem 1, an auxiliary sequence $y_{t}$ is introduced as follows:

(A1) $$\begin{array}{c} y_{t}={\bar{x}}_{t}+\frac{\beta_{1}}{1-\beta_{1}}\left({\bar{x}}_{t}-{\bar{x}}_{t-1}-\frac{\nu (1-\beta_{1})}{\beta_{1}}{\bar{g}}_{t-1}\right), \end{array}$$

where ${\bar{x}}_{t}$ and ${\bar{g}}_{t}$ are averages of local model $x_{t}^{i}$ and stochastic gradient $g_{t}^{i}$ across *n* clients from Algorithm 1, respectively, and we define ${\bar{x}}_{0}={\bar{x}}_{-1}$, ${\bar{g}}_{-1}=0$. Similarly, we define the average vectors ${\bar{m}}_{t}=\frac{1}{n}\sum_{i=1}^{n}m_{t}^{i}$ and ${\bar{d}}_{t}=\frac{1}{n}\sum_{i=1}^{n}d_{t}^{i}$. Applying the update rule of Algorithm 1 implies that:

(A2) $$\begin{array}{c} {\bar{m}}_{t}=\beta_{1}{\bar{m}}_{t-1}+\left(1-\beta_{1}\right){\bar{g}}_{t-1}, \end{array}$$

(A3) $$\begin{array}{c} {\bar{d}}_{t}=\beta_{1}{\bar{d}}_{t-1}+\left(1-\beta_{1}\right)\left({\bar{g}}_{t-1}-{\bar{g}}_{t-2}\right), \end{array}$$

(A4) $$\begin{array}{c} {\bar{x}}_{t}={\bar{x}}_{t-1}-\frac{\eta }{\sqrt{{\hat{v}}_{t}}}⊙{\bar{m}}_{t}+\nu {\bar{d}}_{t}. \end{array}$$

In order to prove Theorem 1, the following lemmas are needed. We defer to their proofs in Appendix A.2.

**Lemma A1.** 
*For sequence $y_{t}$ defined in (A1), we have:*

(A5)
$$y_{t+1}-y_{t}=\frac{\eta \beta_{1}}{1-\beta_{1}}\left(\frac{1}{\sqrt{{\hat{v}}_{t}}}-\frac{1}{\sqrt{{\hat{v}}_{t+1}}}\right)⊙{\bar{m}}_{t}-\eta \frac{{\bar{g}}_{t}}{\sqrt{{\hat{v}}_{t+1}}}.$$

From Lemma A1, we connect $y_{t+1}-y_{t}$ with the two terms on the right-hand side of (A5). The following two lemmas give bounds of distance of $∥y_{t}-{\bar{x}}_{t}{∥}^{2}$ and $\frac{1}{n}\sum_{i=1}^{n}{∥{\bar{x}}_{t}-x_{t}^{i}∥}^{2}$, respectively.

**Lemma A2.** 
*For sequence $y_{t}$ and average of model ${\bar{x}}_{t}$, we have:*

(A6)
$$∥y_{t}-{\bar{x}}_{t}{∥}^{2}\leq 2\left(\frac{\eta^{2}\beta_{1}^{2}}{{\left(1-\beta_{1}\right)}^{2}\delta }+\nu^{2}\right)dG_{\infty }^{2}.$$

**Lemma A3.** 
*For ${\bar{x}}_{t}$ defined in (A4), the sequence of iterations $x_{t}^{i}$ generated by Algorithm 1, for t≥1, we have:*

(A7)
$$\frac{1}{n}\sum_{i=1}^{n}{∥{\bar{x}}_{t}-x_{t}^{i}∥}^{2}\leq 2\left(\frac{\eta^{2}}{\delta }+\frac{4\nu^{2}{\left(1-\beta_{1}\right)}^{2}}{\beta_{1}^{2}}\right)E^{2}dG_{\infty }^{2}.$$

Following Lemma A3, the corresponding average drift bound for $\frac{1}{n}\sum_{i=1}^{n}{∥{\bar{x}}_{t}-x_{t}^{i}∥}^{2}$ is derived in the following Lemma.

**Lemma A4.** 
*Under Algorithm 1, the following relations hold:*

(A8)
$$\sum_{t=1}^{T}{∥\left(\frac{1}{\sqrt{{\hat{v}}_{t}}}-\frac{1}{\sqrt{{\hat{v}}_{t+1}}}\right)⊙{\bar{m}}_{t}∥}^{2}\leq 2G_{\infty }^{2}\frac{d}{\delta },$$

(A9)
$$E{∥\frac{{\bar{g}}_{t}}{\sqrt{{\hat{v}}_{t+1}}}∥}^{2}\leq {∥\frac{\nabla \bar{f}\left(x_{t}\right)}{\sqrt{{\hat{v}}_{t+1}}}∥}^{2}+\frac{d\sigma^{2}}{n\delta }.$$

From Lemma A4, we can provide bounds on $∥y_{t+1}-y_{t}{∥}^{2}$, which play an important role in the proof of the Theorem. Finally, with the previous Lemmas, we return to prove Theorem 1.

**Proof of Theorem 1.** Due to the smoothness of *f*, for t≥0, we have:

(A10) $$\begin{array}{cc} Ef\left(y_{t+1}\right)-Ef\left(y_{t}\right) & \leq E\langle \nabla f\left(y_{t}\right),y_{t+1}-y_{t}\rangle +\frac{L}{2}E{∥y_{t+1}-y_{t}∥}^{2}. \end{array}$$

Summing over *t* from 0 to T−1, and by Lemma 1, we have:

(A11) Ef(yT)−Ef(y0)≤ηβ11−β1∑t=0T−1E∇f(yt),(1v^t−1v^t+1)⊙m¯t=−η∑t=0T−1E∇f(yt),∇f¯(xt)v^t+1+∑t=0T−1Lη2g¯tv^t+12=+∑t=0T−1Lηβ11−β121v^t−1v^t+1)⊙m¯t2,

where $\nabla \bar{f}\left(x_{t}\right)=\frac{1}{n}\sum_{i=1}^{n}\nabla f_{i}\left(x_{t}^{i}\right)$ and $Eg_{t}^{i}=\nabla f_{i}\left(x_{t}^{i}\right)$ is used from Assumption 3. We next bound the terms on the right-hand side of (A11). By (A27) and the non-decreasing property of ${\hat{v}}_{t}$, we have:

(A12) $$\begin{array}{cc} \; & \sum_{t=0}^{T-1}\frac{\eta \beta_{1}}{1-\beta_{1}}E\left[\nabla f{\left(y_{t}\right)}^{⊤}\left(\frac{1}{\sqrt{{\hat{v}}_{t}}}-\frac{1}{\sqrt{{\hat{v}}_{t+1}}}\right)⊙{\bar{m}}_{t}\right]\\ \; & \leq \frac{\eta \beta_{1}}{1-\beta_{1}}G_{\infty }^{2}E\left[\sum_{t=0}^{T-1}\sum_{j=1}^{d}{\left(\frac{1}{\sqrt{{\hat{v}}_{t}}}-\frac{1}{\sqrt{{\hat{v}}_{t+1}}}\right)}_{j}\right]\\ \; & \leq \frac{\eta \beta_{1}}{1-\beta_{1}}G_{\infty }^{2}E\left[\sum_{j=1}^{d}{\left(\frac{1}{\sqrt{{\hat{v}}_{0}}}-\frac{1}{\sqrt{{\hat{v}}_{T}}}\right)}_{j}\right]\\ \; & \leq \frac{\eta \beta_{1}}{1-\beta_{1}}G_{\infty }^{2}\frac{d}{\sqrt{\delta }}. \end{array}$$

By the equality $\langle a,b\rangle =\frac{1}{2}{(∥a∥}^{2}+{∥b∥}^{2}{-∥a-b∥}^{2})$, we have:

(A13) −ηE∇f(yt),∇f¯(xt)v^t+1=η2E−∇f(yt)(v^t+1)142=−∇f¯(xt)(v^t+1)142+∇f(yt)−∇f¯(xt)(v^t+1)142.

Furthermore, we bound the last term of (A13). By the smoothness of $f_{i}\left(x\right)$, together with (A6) and (A7), we have:

(A14) η2∇f(yt)−∇f¯(xt)(v^t+1)142=1n∑i=1n∇fi(yt)−∇fi(xti)(v^t+1)1/42≤ηn∑i=1n∇fi(yt)−∇fi(x¯t)(v^t+1)1/42+∇fi(x¯t)−∇fi(xti)(v^t+1)1/42≤ηn∑i=1nL2yt−x¯t2+x¯t−xti2minj∈[d](v^t+1)j1/2≤2ηL2dG∞2minj∈[d](v^t+1)j1/2η2δβ12(1−β1)2+E2=+ν24(1−β1)2β12E2+1.

Plugging (A8), (A9), (A12) and (A14) into (A11), we obtain:

(A15) Ef(yT)−Ef(y0)≤−η2∑t=0T−1E∇f(yt)(v^t+1)142+E∇f¯(xt)(v^t+1)142=+η2L∑t=0T−1∇f¯(xt)v^t+12+β12dG∞2(1−β1)2δ+dσ2nδ=+2ηL2dG∞2minj∈[d](v^t+1)j1/2∑t=0T−1η2δβ12(1−β1)2+E2=+ν24(1−β1)2β12E2+1+ηβ1dG∞2(1−β1)δ.

Dividing by ηT both sides of (A15), and rearranging the terms, we have:

(A16) 12T∑t=0T−1E∇f(yt)(v^t+1)142+E∇f¯(xt)(v^t+1)142≤1ηTEf(y0)−Ef(yT)+ηLT∑t=0T−1E∇f¯(xt)v^t+12=+β12dG∞2(1−β1)2δ+dσ2nδ+β1T(1−β1)dG∞2δ=+2L2dG∞2Tminj∈[d](v^t+1)j1/2∑t=0T−1η2δβ12(1−β1)2+E2=+ν24(1−β1)2β12E2+1.

Furthermore, choosing $\eta =\min\{\frac{\sqrt{\delta }}{4L},\sqrt{\frac{n}{Td}}\}$ yields:

(A17) $$\begin{array}{cc} \frac{\eta L}{T}\sum_{t=0}^{T-1}E{∥\frac{\nabla \bar{f}(x_{t})}{\sqrt{{\hat{v}}_{t+1}}}∥}^{2} & \leq \frac{\eta L}{T}\sum_{i=0}^{T-1}E\left[\sum_{j=1}^{d}\frac{1}{\sqrt{\delta }}\frac{\left(\nabla \bar{f}(x_{t}\right){)}_{j}^{2}}{{\left({\hat{v}}_{t+1}^{1/4}\right)}_{j}^{2}}\right]\\ \; & \leq \frac{\eta L}{T}\frac{1}{\sqrt{\delta }}\sum_{i=0}^{T-1}E{∥\frac{\nabla \bar{f}(x_{t})}{{\hat{v}}_{t+1}^{1/4}}∥}^{2}\\ \; & \leq \frac{1}{4T}\sum_{t=0}^{T-1}E{∥\frac{\nabla \bar{f}(x_{t})}{{\hat{v}}_{t+1}^{1/4}}∥}^{2}. \end{array}$$

Plugging (A17) into (A16), together with $T\geq \frac{16nL^{2}}{d\delta }$ and $\nu =\frac{1}{\sqrt{Td}}$, we have:

(A18) 12T∑t=0T−1E∇f(yt)v^t+11/42+14T∑t=0T−1E∇f¯(xt)v^t+11/42≤dnTEf(y0)−Ef(yT)+dnTLσ2δ+dTβ11−β1G∞2δ=+d4Tβ12(1−β1)2G∞2δ+2nTL2G∞2δ3/2β12(1−β1)2+E2=+2TL2G∞2δ4(1−β1)2β12E2+1.

Further, by Cauchy–Schwarz inequality, we have:

(A19) $$\begin{array}{cc} {∥\frac{\nabla \bar{f}\left(x_{t}\right)}{{\hat{v}}_{t+1}^{1/4}}∥}^{2} & \geq \frac{1}{2}{∥\frac{\nabla f({\bar{x}}_{t})}{{\hat{v}}_{t+1}^{1/4}}∥}^{2}-{∥\frac{\nabla f\left({\bar{x}}_{t}\right)-\nabla \bar{f}\left(x_{t}\right)}{{\hat{v}}_{t+1}^{1/4}}∥}^{2}\\ \; & \geq \frac{1}{2}{∥\frac{\nabla f({\bar{x}}_{t})}{{\hat{v}}_{t+1}^{1/4}}∥}^{2}-\frac{1}{n}\sum_{i=1}^{n}∥\frac{\nabla f_{i}\left({\bar{x}}_{t}\right)-\nabla f_{i}\left(x_{t}^{i}\right)}{{\hat{v}}_{t+1}^{1/4}}∥\\ \; & \geq \frac{1}{2}{∥\frac{\nabla f({\bar{x}}_{t})}{{\hat{v}}_{t+1}^{1/4}}∥}^{2}-\frac{2L^{2}E^{2}dG_{\infty }^{2}}{\sqrt{\delta }}\left(\frac{\eta^{2}}{\delta }+\frac{4\nu^{2}{\left(1-\beta_{1}\right)}^{2}}{\beta_{1}^{2}}\right), \end{array}$$

where the second inequality is because of the Jensen’s inequality, and the last is from the smoothness of $f_{i}$ and Lemma A3. Plugging (A19) into (A18) and multiplying both sides of this inequality by 8 yields:

(A20) 1T∑t=0T−1E∇f(x¯t)v^t+11/42≤8dnTf(x¯0)−f∗+2dnTLσ2δ+8dTβ11−β1G∞2δ=+2dTβ12(1−β1)2G∞2δ+16nTL2G∞2δ3/2β12(1−β1)2+5E24=+16TL2G∞2δ5(1−β1)2β12E2+1,

where $y_{0}={\bar{x}}_{0}$ and $f^{*}=f\left(x^{*}\right)$ is the minimum value at the optimal point $x^{*}$. Hence, we complete the proof. □

### Appendix A.2. Proof of Lemmas

**Proof of Lemma A1.** From the definition of $y_{t}$, we have:

yt+1−yt=11−β1(x¯t+1−x¯t)−β11−β1(x¯t−x¯t−1)−ν(g¯t−g¯t−1)=11−β1−ηv^t+1⊙m¯t+1+νd¯t+1−νg¯t−g¯t−1=−β11−β1−ηv^t⊙m¯t+νd¯t.

According to (A2) and (A3), we have:

$$\begin{array}{cc} y_{t+1}-y_{t} & =\frac{\eta \beta_{1}}{1-\beta_{1}}\left(\frac{1}{\sqrt{{\hat{v}}_{t}}}-\frac{1}{\sqrt{{\hat{v}}_{t+1}}}\right)⊙{\bar{m}}_{t}-\frac{\eta }{\sqrt{{\hat{v}}_{t+1}}}⊙{\bar{g}}_{t}, \end{array}$$

which finishes the proof. □

**Proof of Lemma A2.** Recalling (A1) and (A4), we have:

(A21) $$\begin{array}{cc} y_{t}-{\bar{x}}_{t} & =-\frac{\beta_{1}}{1-\beta_{1}}\frac{\eta }{\sqrt{{\hat{v}}_{t}}}⊙{\bar{m}}_{t}+\frac{\nu \beta_{1}}{1-\beta_{1}}{\bar{d}}_{t}-\nu {\bar{g}}_{t-1}. \end{array}$$

Iteratively using recursion for Equation (A3), together with ${\bar{d}}_{0}=0$, we can obtain:

(A22) $${\bar{d}}_{t}=\left(1-\beta_{1}\right)\sum_{j=0}^{t-1}\beta_{1}^{t-1-j}\left({\bar{g}}_{j}-{\bar{g}}_{j-1}\right).$$

We then expand ${\bar{d}}_{t}$ in (A22), noting that ${\bar{g}}_{-1}=0$:

(A23) $$\begin{array}{cc} {\bar{d}}_{t} & =\left(1-\beta_{1}\right)\left(\sum_{j=0}^{t-1}\beta_{1}^{t-1-j}{\bar{g}}_{j}-\sum_{j=1}^{t-1}\beta_{1}^{t-1-j}{\bar{g}}_{j-1}\right)\\ \; & =\left(1-\beta_{1}\right)\left({\bar{g}}_{t-1}-\left(1-\beta_{1}\right)\sum_{j=1}^{t-1}\beta_{1}^{t-1-j}{\bar{g}}_{j-1}\right)\\ \; & =\left(1-\beta_{1}\right)\left({\bar{g}}_{\left(t-1\right)}+\frac{1-\beta_{1}}{\beta_{1}}{\bar{g}}_{t-1}-\left(1-\beta_{1}\right)\sum_{j=0}^{t-1}\beta_{1}^{t-2-j}{\bar{g}}_{j}\right)\\ \; & =\frac{1-\beta_{1}}{\beta_{1}}\left({\bar{g}}_{\left(t-1\right)}-\left(1-\beta_{1}\right)\sum_{j=0}^{t-1}\beta_{1}^{t-1-j}{\bar{g}}_{j}\right). \end{array}$$

For t≥1, substituting (A23) into (A21) yields:

(A24) $$y_{t}-{\bar{x}}_{t}=-\frac{\beta_{1}}{1-\beta_{1}}\frac{\eta }{\sqrt{{\hat{v}}_{t}}}⊙{\bar{m}}_{t}-\nu \left(1-\beta_{1}\right)\sum_{j=0}^{t-1}\beta_{1}^{t-1-j}{\bar{g}}_{j}.$$

Furthermore, we measure the difference between $y_{t}$ and ${\bar{x}}_{t}$. Using inequality ${∥a+b∥}^{2}\leq {2∥a∥}^{2}+2{∥b∥}^{2}$ implies that:

(A25) $$\begin{array}{cc} ∥y_{t}-{\bar{x}}_{t}{∥}^{2} & \leq \frac{2\eta^{2}\beta_{1}^{2}}{{\left(1-\beta_{1}\right)}^{2}}{∥\frac{{\bar{m}}_{t}}{\sqrt{{\hat{v}}_{t}}}∥}^{2}+2\nu^{2}{\left(1-\beta_{1}\right)}^{2}{∥\sum_{j=0}^{t-1}\beta_{1}^{t-1-j}{\bar{g}}_{j}∥}^{2}. \end{array}$$

$∥m_{t}^{i}{∥}_{\infty }$ is first estimated by induction. In fact, by Assumption 2, the stochastic gradient has bounded the $l_{\infty }$ norm, and we have:

(A26) $$\begin{array}{c} ∥{\bar{g}}_{t}∥=∥\frac{1}{n}\sum_{i=1}^{n}g_{t}^{i}∥\leq \frac{1}{n}\sum_{i=1}^{n}∥g_{t}^{i}∥\leq \sqrt{d}G_{\infty }, \end{array}$$

where *d* is the dimensionality of vectors. For $m_{t}^{i}$, since ${∥m_{0}^{i}∥}_{\infty }=0$, suppose that ${∥m_{t}^{i}∥}_{\infty }\leq G_{\infty }$; then:

(A27) $$\begin{array}{cc} ∥m_{t+1}^{i}{∥}_{\infty } & ={∥\beta_{1}m_{t}^{i}+\left(1-\beta_{1}\right)g_{t}^{i}∥}_{\infty }\\ \; & \leq \beta_{1}∥m_{t}^{i}{∥}_{\infty }+\left(1-\beta_{1}\right){∥g_{t}^{i}∥}_{\infty }\\ \; & \leq \beta_{1}G_{\infty }+\left(1-\beta_{1}\right)G_{\infty }\\ \; & =G_{\infty }. \end{array}$$

Thus, according to the definition of norm, we have:

(A28) $$\begin{array}{cc} {∥\frac{{\bar{m}}_{t}}{{\sqrt{\hat{v}}}_{t}}∥}^{2} & =\sum_{j=1}^{d}\frac{{\left(\frac{1}{n}\sum_{i=1}^{n}m_{t}^{i}\right)}_{j}^{2}}{{\left(\sqrt{{\hat{v}}_{t}}\right)}_{j}^{2}}\leq \frac{1}{n}\sum_{j=1}^{d}\frac{\sum_{i=1}^{n}{\left(m_{t}^{i}\right)}_{j}^{2}}{\delta }\leq \frac{dG_{\infty }^{2}}{\delta }, \end{array}$$

where subscripted *j* denotes the *j*-th component of vectors. The first inequality is from the non-decreasing nature of ${\hat{v}}_{t}$ and ${\left({\hat{v}}_{t}\right)}_{j}\geq \delta$, for any j∈{1,⋯,d}. Define $s_{t}≜\sum_{j=0}^{t-1}\beta_{1}^{t-1-j}=\frac{1-\beta_{1}^{t}}{1-\beta_{1}}$. Because of the convexity of $l_{2}$ norm and Jensen’s inequality, we have:

(A29) $$\begin{array}{cc} {∥\sum_{j=0}^{t-1}\beta_{1}^{t-1-j}{\bar{g}}_{j}∥}^{2} & =s_{t}^{2}{∥\sum_{j=0}^{t-1}\frac{\beta_{1}^{t-1-j}}{s_{t}}{\bar{g}}_{j}∥}^{2}\leq s_{t}\sum_{j=0}^{t-1}\beta_{1}^{t-1-j}{∥{\bar{g}}_{j}∥}^{2}\leq \frac{dG_{\infty }^{2}}{{\left(1-\beta_{1}\right)}^{2}}. \end{array}$$

Substituting (A28) and (A29) into (A25), we finish the proof. □

**Proof of Lemma A3.** When *t* is an aggregation moment, i.e., a multiple of *E*, it is easy to see ${\bar{x}}_{t}-x_{t}^{i}=0$. Thus, we focus on studying the upper bounds of $∥{\bar{x}}_{t}-x_{t}^{i}∥$ when *t* is not a multiple of *E*. Let $t_{0}<t$ be the largest number of iteration that is an aggregation moment, and $t-t_{0}<E$. According to the update rule (5), we have:

(A30) $$\begin{array}{c} x_{t}^{i}=x_{t_{0}}^{i}-\sum_{h=t_{0}+1}^{t}\frac{\eta }{\sqrt{{\hat{v}}_{h}}}⊙m_{h}^{i}+\nu \sum_{l=t_{0}+1}^{t}d_{l}^{i}. \end{array}$$

Summing over i∈{1,⋯,n}, we further obtain:

(A31) $$\begin{array}{cc} {\bar{x}}_{t} & ={\bar{x}}_{t_{0}}-\frac{1}{n}\sum_{i=1}^{n}\sum_{h=t_{0}+1}^{t}\frac{\eta }{\sqrt{{\hat{v}}_{h}}}⊙m_{h}^{i}+\frac{\nu }{n}\sum_{i=1}^{n}\sum_{l=t_{0}+1}^{t}d_{l}^{i}. \end{array}$$

Combing (A30) and (A31), and noticing ${\bar{x}}_{t_{0}}=x_{t_{0}}^{i}$, it can be verified that:

(A32) 1n∑i=1nx¯t−xti2=1n∑i=1nη∑h=t0+1t(mhiv^h−1n∑i=1nmhiv^h)+ν∑l=t0+1t(1n∑i=1ndli−dli)2≤2n∑i=1nη2∑h=t0+1tmhiv^h−1n∑i=1n∑h=t0+1tmhiv^h2=+2n∑i=1nν2∑l=t0+1tdli−1n∑i=1n∑l=t0+1tdli2,

where the inequality follows from ${∥a+b∥}^{2}\leq {2∥a∥}^{2}+2{∥b∥}^{2}$. We next bound the two terms on the right-hand side of (A32), respectively. By $\frac{1}{n}\sum_{i=1}^{n}{∥a^{i}-\left(\frac{1}{n}\sum_{j=1}^{n}a^{j}\right)∥}^{2}=\frac{1}{n}\sum_{i=1}^{n}{∥a^{i}∥}^{2}-{∥\frac{1}{n}\sum_{i=1}^{n}a^{i}∥}^{2}\leq \frac{1}{n}\sum_{i=1}^{n}{∥a^{i}∥}^{2}$ with $a^{i}=\sum_{h=t_{0}+1}^{t}\frac{m_{h}^{i}}{\sqrt{{\hat{v}}_{h}}}$, it can be verified that:

(A33) $$\begin{array}{cc} \frac{2}{n}\sum_{i=1}^{n}\eta^{2}{∥\sum_{h=t_{0}+1}^{t}\frac{m_{h}^{i}}{\sqrt{{\hat{v}}_{h}}}-\frac{1}{n}\sum_{i=1}^{n}\sum_{h=t_{0}+1}^{t}\frac{m_{h}^{i}}{\sqrt{{\hat{v}}_{h}}}∥}^{2} & \leq \frac{2}{n}\sum_{i=1}^{n}\eta^{2}{∥\sum_{h=t_{0}+1}^{t}\frac{m_{h}^{i}}{\sqrt{{\hat{v}}_{h}}}∥}^{2}\\ \; & \leq \frac{2}{n}\sum_{i=1}^{n}\eta^{2}\left(t-t_{0}\right)\sum_{h=t_{0}+1}^{t}{∥\frac{m_{h}^{i}}{\sqrt{{\hat{v}}_{h}}}∥}^{2}\\ \; & \leq 2\eta^{2}E^{2}d\frac{G_{\infty }^{2}}{\delta }, \end{array}$$

where the second inequality is because $t-t_{0}<E$ and ${\left({\hat{v}}_{h}\right)}_{j}\geq \delta$, for ∀j∈{1,⋯,d} and (A27). Similarly, it can be proved that:

(A34) $$\begin{array}{cc} \; & \frac{2}{n}\sum_{i=1}^{n}\nu^{2}{∥\sum_{l=t_{0}+1}^{t}d_{l}^{i}-\frac{1}{n}\sum_{i=1}^{n}\sum_{l=t_{0}+1}^{t}d_{l}^{i}∥}^{2}\\ \; & \leq \frac{2}{n}\sum_{i=1}^{n}\nu^{2}\left(t-t_{0}\right)\sum_{l=t_{0}+1}^{t}{∥d_{l}^{i}∥}^{2}\\ \; & \leq 8\nu^{2}E^{2}\frac{{\left(1-\beta_{1}\right)}^{2}}{\beta_{1}^{2}}dG_{\infty }^{2}, \end{array}$$

where the last inequality is because:

(A35) $$\begin{array}{cc} ∥d_{l}^{i}∥ & =∥\frac{1-\beta_{1}}{\beta_{1}}\left(g_{t-1}^{i}-\left(1-\beta_{1}\right)\sum_{j=0}^{t-1}\beta_{1}^{j}g_{t-1-j}^{i}\right)∥\\ \; & \leq \frac{1-\beta_{1}}{\beta_{1}}\left(∥g_{t-1}^{i}∥+\left(1-\beta_{1}\right)∥\sum_{j=0}^{t-1}\beta_{1}^{j}g_{t-1-j}^{i}∥\right)\\ \; & \leq \frac{2(1-\beta_{1})}{\beta_{1}}\sqrt{d}G_{\infty }. \end{array}$$

Substituting (A33) and (A34) into (A32), we finish the proof. □

**Proof of Lemma A4.** According to (A5), it is easy to know that:

(A36) $$\begin{array}{cc} ∥y_{t+1}-y_{t}{∥}^{2} & ={∥\frac{\eta \beta_{1}}{1-\beta_{1}}\left(\frac{1}{\sqrt{{\hat{v}}_{t}}}-\frac{1}{\sqrt{{\hat{v}}_{t+1}}}\right)⊙{\bar{m}}_{t}-\eta \frac{{\bar{g}}_{t}}{\sqrt{{\hat{v}}_{t+1}}}∥}^{2}\\ \; & \leq \frac{2\eta^{2}\beta_{1}^{2}}{{\left(1-\beta_{1}\right)}^{2}}{∥\left(\frac{1}{\sqrt{{\hat{v}}_{t}}}-\frac{1}{\sqrt{{\hat{v}}_{t+1}}}\right)⊙{\bar{m}}_{t}∥}^{2}+2\eta^{2}{∥\frac{{\bar{g}}_{t}}{\sqrt{{\hat{v}}_{t+1}}}∥}^{2}. \end{array}$$

Now, we estimate the two terms on the right-hand side of (A36). Applying the definition of norm implies that:

(A37) $$\begin{array}{cc} \sum_{t=1}^{T}{∥\left(\frac{1}{\sqrt{{\hat{v}}_{t}}}-\frac{1}{\sqrt{{\hat{v}}_{t+1}}}\right)⊙{\bar{m}}_{t}∥}^{2} & =\sum_{t=1}^{T}\sum_{j=1}^{d}{\left({\left(\frac{1}{\sqrt{{\hat{v}}_{t}}}-\frac{1}{\sqrt{{\hat{v}}_{t+1}}}\right)}_{j}{\left(\frac{1}{n}\sum_{i=1}^{n}m_{t}^{i}\right)}_{j}\right)}^{2}\\ \; & \leq \sum_{t=1}^{T}G_{\infty }^{2}\sum_{j=1}^{d}\frac{1}{\sqrt{\delta }}\left|\frac{1}{{\left(\sqrt{{\hat{v}}_{t}}\right)}_{j}}-\frac{1}{{\left(\sqrt{{\hat{v}}_{t+1}}\right)}_{j}}\right|\\ \; & \leq G_{\infty }^{2}\frac{1}{\sqrt{\delta }}\sum_{j=1}^{d}\left|\frac{1}{{\left(\sqrt{{\hat{v}}_{1}}\right)}_{j}}-\frac{1}{{\left(\sqrt{{\hat{v}}_{T+1}}\right)}_{j}}\right|\\ \; & \leq \frac{dG_{\infty }^{2}}{\delta }, \end{array}$$

where the first inequality follows from (A27) and the non-decreasing nature of ${\hat{v}}_{t}$. Additionally, taking expectation, together with the definition of variance, we have:

(A38) $$\begin{array}{cc} E{∥\frac{{\bar{g}}_{t}}{\sqrt{{\hat{v}}_{t+1}}}∥}^{2}\; & =E{∥\frac{\frac{1}{n}\sum_{i=1}^{n}g_{t}^{i}-\frac{1}{n}\sum_{i=1}^{n}\nabla f_{i}\left(x_{t}^{i}\right)}{\sqrt{{\hat{v}}_{t+1}}}∥}^{2}+{∥E\frac{\frac{1}{n}\sum_{i=1}^{n}g_{t}^{i}}{\sqrt{{\hat{v}}_{t+1}}}∥}^{2}\\ \; & \leq \frac{1}{n^{2}}\sum_{i=1}^{n}E{∥\frac{g_{t}^{i}-\nabla f_{i}\left(x_{t}^{i}\right)}{\sqrt{{\hat{v}}_{t+1}}}∥}^{2}+{∥\frac{\nabla \bar{f}\left(x_{t}\right)}{\sqrt{{\hat{v}}_{t+1}}}∥}^{2}\\ \; & \leq {∥\frac{\nabla \bar{f}\left(x_{t}\right)}{\sqrt{{\hat{v}}_{t+1}}}∥}^{2}+\frac{d\sigma^{2}}{n\delta }, \end{array}$$

where $\nabla \bar{f}\left(x_{t}\right)=\frac{1}{n}\sum_{i=1}^{n}\nabla f_{i}\left(x_{t}^{i}\right)$ is the gradient averages of clients at local solutions, and the last inequality follows that the coordination-wise of stochastic gradients has σ-bounded variance from Assumption 3 and ${\left({\hat{v}}_{t}\right)}_{j}\geq \delta$ for ∀j∈{1,⋯,d},∀t≥0. This completes the proof. □

## References

[1] Konečný J., McMahan H.B., Yu F.X., Richtárik P., Suresh A.T., Bacon D. (2016). Federated Learning: Strategies for Improving Communication Efficiency. arXiv.

[2] McMahan B., Moore E., Ramage D., Hampson S., y Arcas B.A. Communication-Efficient Learning of Deep Networks from Decentralized Data. Proceedings of the 20th International Conference on Artificial Intelligence and Statistics.

[3] Yang Y., Hong Y., Park J. (2021). Efficient Gradient Updating Strategies with Adaptive Power Allocation for Federated Learning over Wireless Backhaul. Sensors.

[4] Yang W., Zhang Y., Ye K., Li L., Xu C. FFD: A Federated Learning Based Method for Credit Card Fraud Detection. Proceedings of the Big Data—BigData 2019—8th International Congress.

[5] Xu J., Glicksberg B.S., Su C., Walker P.B., Bian J., Wang F. (2021). Federated Learning for Healthcare Informatics. J. Healthc. Inform. Res..

[6] Stich S.U. Local SGD Converges Fast and Communicates Little. Proceedings of the 7th International Conference on Learning Representations.

[7] Wang J., Joshi G. (2018). Cooperative SGD: A unified Framework for the Design and Analysis of Communication-Efficient SGD Algorithms. arXiv.

[8] Khaled A., Mishchenko K., Richtárik P. Tighter Theory for Local SGD on Identical and Heterogeneous Data. Proceedings of the 23rd International Conference on Artificial Intelligence and Statistics.

[9] Yu H., Jin R., Yang S. On the Linear Speedup Analysis of Communication Efficient Momentum SGD for Distributed Non-Convex Optimization. Proceedings of the 36th International Conference on Machine Learning.

[10] Li T., Sahu A.K., Zaheer M., Sanjabi M., Talwalkar A., Smith V. Federated Optimization in Heterogeneous Networks. Proceedings of the Proceedings of Machine Learning and Systems.

[11] Karimireddy S.P., Kale S., Mohri M., Reddi S.J., Stich S.U., Suresh A.T. SCAFFOLD: Stochastic Controlled Averaging for Federated Learning. Proceedings of the 37th International Conference on Machine Learning.

[12] Liu W., Chen L., Chen Y., Zhang W. (2020). Accelerating Federated Learning via Momentum Gradient Descent. IEEE Trans. Parallel Distrib. Syst..

[13] Ozfatura E., Ozfatura K., Gündüz D. FedADC: Accelerated Federated Learning with Drift Control. Proceedings of the IEEE International Symposium on Information Theory.

[14] Reddi S.J., Charles Z., Zaheer M., Garrett Z., Rush K., Konečný J., Kumar S., McMahan H.B. Adaptive Federated Optimization. Proceedings of the 9th International Conference on Learning Representations.

[15] Khanduri P., Sharma P., Yang H., Hong M., Liu J., Rajawat K., Varshney P.K. STEM: A Stochastic Two-Sided Momentum Algorithm Achieving Near-Optimal Sample and Communication Complexities for Federated Learning. Proceedings of the Advances in Neural Information Processing Systems.

[16] Xu A., Huang H. (2021). Double Momentum SGD for Federated Learning. arXiv.

[17] Chen X., Li X., Li P. Toward Communication Efficient Adaptive Gradient Method. Proceedings of the FODS’20: ACM-IMS Foundations of Data Science Conference.

[18] Wang Y., Lin L., Chen J. Communication-Efficient Adaptive Federated Learning. Proceedings of the International Conference on Machine Learning.

[19] Ogata K. (2010). Modern Control Engineering.

[20] Ma R., Zhang B., Zhou Y., Li Z., Lei F. (2022). PID Controller-Guided Attention Neural Network Learning for Fast and Effective Real Photographs Denoising. IEEE Trans. Neural Netw. Learn. Syst..

[21] An W., Wang H., Sun Q., Xu J., Dai Q., Zhang L. A PID Controller Approach for Stochastic Optimization of Deep Networks. Proceedings of the IEEE Conference on Computer Vision and Pattern Recognition.

[22] Shi L., Zhang Y., Wang W., Cheng J., Lu H. Rethinking The Pid Optimizer For Stochastic Optimization Of Deep Networks. Proceedings of the IEEE International Conference on Multimedia and Expo.

[23] Recht B. (2019). A tour of reinforcement learning: The view from continuous control. Annu. Rev. Control Robot. Auton. Syst..

[24] Polyak B.T. (1964). Some methods of speeding up the convergence of iteration methods. USSR Comput. Math. Math. Phys..

[25] Nesterov Y. (1983). A method of solving a convex programming problem with convergence rate O (1/*k*^2^). Sov. Math. Dokl..

[26] Goodfellow I.J., Bengio Y., Courville A.C. (2016). Deep Learning.

[27] Sutskever I., Martens J., Dahl G.E., Hinton G.E. On the importance of initialization and momentum in deep learning. Proceedings of the 30th International Conference on Machine Learning.

[28] Liu D., Lu Z. (2021). The Convergence Rate of SGD’s Final Iterate: Analysis on Dimension Dependence. arXiv.

[29] Amir I., Koren T., Livni R. SGD Generalizes Better Than GD (And Regularization Doesn’t Help). Proceedings of the Conference on Learning Theory.

[30] Duchi J.C., Hazan E., Singer Y. (2011). Adaptive Subgradient Methods for Online Learning and Stochastic Optimization. J. Mach. Learn. Res..

[31] Kingma D.P., Ba J. Adam: A Method for Stochastic Optimization. Proceedings of the International Conference on Learning Representations.

[32] Reddi S.J., Kale S., Kumar S. On the Convergence of Adam and Beyond. Proceedings of the International Conference on Learning Representations.

[33] Zaheer M., Reddi S.J., Sachan D.S., Kale S., Kumar S. Adaptive Methods for Nonconvex Optimization. Proceedings of the Advances in Neural Information Processing Systems.

[34] Zhuang J., Tang T., Ding Y., Tatikonda S.C., Dvornek N.C., Papademetris X., Duncan J.S. AdaBelief Optimizer: Adapting Stepsizes by the Belief in Observed Gradients. Proceedings of the Advances in Neural Information Processing Systems.

[35] Prazeres M.O., Oberman A.M. (2021). Stochastic Gradient Descent with Polyak’s Learning Rate. J. Sci. Comput..

[36] Loizou N., Vaswani S., Laradji I.H., Lacoste-Julien S. Stochastic Polyak Step-size for SGD: An Adaptive Learning Rate for Fast Convergence. Proceedings of the 24th International Conference on Artificial Intelligence and Statistics.

[37] Berrada L., Zisserman A., Kumar M.P. Training Neural Networks for and by Interpolation. Proceedings of the 37th International Conference on Machine Learning.

[38] Glasgow M.R., Yuan H., Ma T. Sharp Bounds for Federated Averaging (Local SGD) and Continuous Perspective. Proceedings of the 25th International Conference on Artificial Intelligence and Statistics.

[39] Gorbunov E., Hanzely F., Richtárik P. Local SGD: Unified Theory and New Efficient Methods. Proceedings of the 24th International Conference on Artificial Intelligence and Statistics.

[40] Guo Y., Sun Y., Hu R., Gong Y. Hybrid Local SGD for Federated Learning with Heterogeneous Communications. Proceedings of the 10th International Conference on Learning Representations.

[41] Zhang T., Song A., Dong X., Shen Y., Ma J. (2022). Privacy-Preserving Asynchronous Grouped Federated Learning for IoT. IEEE Internet Things J..

[42] Zeng T., Semiari O., Chen M., Saad W., Bennis M. (2021). Federated Learning for Collaborative Controller Design of Connected and Autonomous Vehicles. Proceedings of the 2021 60th IEEE Conference on Decision and Control (CDC).

[43] Haddadpour F., Kamani M.M., Mokhtari A., Mahdavi M. Federated Learning with Compression: Unified Analysis and Sharp Guarantees. Proceedings of the 24th International Conference on Artificial Intelligence and Statistics.

[44] LeCun Y., Bottou L., Bengio Y., Haffner P. (1998). Gradient-based learning applied to document recognition. Proc. IEEE.

[45] Xiao H., Rasul K., Vollgraf R. (2017). Fashion-MNIST: A Novel Image Dataset for Benchmarking Machine Learning Algorithms. arXiv.

